# Epidemiology, clinical characteristics, and outcome of candidemia in critically ill patients in Germany: a single-center retrospective 10-year analysis

**DOI:** 10.1186/s13613-020-00755-8

**Published:** 2020-10-16

**Authors:** Maria Schroeder, Theresa Weber, Timme Denker, Sarah Winterland, Dominic Wichmann, Holger Rohde, Ann-Kathrin Ozga, Marlene Fischer, Stefan Kluge

**Affiliations:** 1 Department of Intensive Care Medicine, University Medical Center Hamburg-Eppendorf, Martinistrasse 52, 20246 Hamburg, Germany; 2 Department of Medical Microbiology, Virology and Hygiene, University Medical Center Hamburg-Eppendorf, Hamburg, Germany; 3 Center for Experimental Medicine, Institute of Medical Biometry and Epidemiology, University Medical Center Hamburg-Eppendorf, Hamburg, Germany

**Keywords:** Candidemia, *Candida* species, Critically ill patients, Intensive care unit, Antifungal agents, Fluconazole, Echinocandin, Bloodstream infection

## Abstract

**Background:**

Despite advances in the management of bloodstream infections (BSI) caused by *Candida *spp., the mortality still remains high in critically ill patients. The worldwide epidemiology of yeast-related BSI is subject to changing species distribution and resistance patterns, challenging antifungal treatment strategies. The aim of this single-center study was to identify predictors of mortality after 28 and 180 days in a cohort of mixed surgical and medical critically ill patients with candidemia.

**Methods:**

Patients, who had been treated for laboratory-confirmed BSI caused by *Candida *spp. in one of 12 intensive care units (ICU) at a University hospital between 2008 and 2017, were retrospectively identified. We retrieved data including clinical characteristics, *Candida* species distribution, and antifungal management from electronic health records to identify risk factors for mortality at 28 and 180 days using a Cox regression model.

**Results:**

A total of 391 patients had blood cultures positive for *Candida *spp. (incidence 4.8/1000 ICU admissions). The mortality rate after 28 days was 47% (*n* = 185) and increased to 60% (*n* = 234) after 180 days. Age (HR 1.02 [95% CI 1.01–1.03]), a history of liver cirrhosis (HR 1.54 [95% CI 1.07–2.20]), septic shock (HR 2.41 [95% CI 1.73–3.37]), the Sepsis-related Organ Failure Assessment score (HR 1.12 [95% CI 1.07–1.17]), Candida score (HR 1.25 [95% CI 1.11–1.40]), and the length of ICU stay at culture positivity (HR 1.01 [95% CI 1.00–1.01]) were significant risk factors for death at 180 days. Patients, who had abdominal surgery (HR 0.66 [95% CI 0.48–0.91]) and patients, who received adequate (HR 0.36 [95% CI 0.24–0.52]) or non-adequate (HR 0.31 [95% CI 0.16–0.62]) antifungal treatment, had a reduced mortality risk compared to medical admission and no antifungal treatment, respectively.

**Conclusions:**

The mortality of critically ill patients with *Candida* BSI is high and is mainly determined by disease severity, multiorgan dysfunction, and antifungal management rather than species distribution and susceptibility. Our results underline the importance of timely treatment of candidemia. However, controversies remain on the optimal definition of adequate antifungal management.

## Background

Bloodstream infections (BSI) caused by *Candida *spp. have become a major concern in critical care medicine due to the increasing number of immunocompromised patients [1, 2]. Yeast-related BSI are usually healthcare-associated infections with a high mortality of up to 60% in critically ill patients [1, 3–5]. Importantly, BSI caused by *Candida *spp., central-line-associated BSI in particular, have been increasing over the past decades [6]. The international EPIC II study including more than 14,000 patients showed that *Candida* was the third most common cause of infection and was second in both North America and Western Europe [7]. Importantly, mortality was found to be substantially higher in patients with *Candida* BSI compared with patients suffering from Gram-positive or Gram-negative BSI [5, 7].

In the past years, a shift in *Candida *spp. towards non-*albicans Candida *spp. has been observed. The proportion of candidemia caused by non-*albicans Candida *spp. was 59.8% in an Italian multicenter survey [8]. An analysis from a North American prospective registry showed similar rates with 57.9% of non-*albicans Candida *spp.[9]. The shift in species distribution may reflect selection pressure induced by prior azole prescription [9–11].

Current recommendations on the management of invasive candidiasis in nonneutropenic patients suggest that adequate source control including catheter removal, should be performed early, if clinically feasible [12]. Although there is strong evidence that a delay in treatment negatively affects outcome, the optimal time point of intervention, such as central venous catheter (CVC) removal, is under debate [13, 14].

The aim of this retrospective study was to identify risk factors for mortality 28 and 180 days after *Candida* BSI. We hypothesized that survival would mainly be determined by appropriate antifungal treatment, such as adequate antifungal substance and timely source control, and yeast spp. Therefore, we analyzed clinical characteristics, antifungal management, and patterns of *Candida *spp. distribution in a mixed population of critically ill patients at 12 multidisciplinary intensive care units (ICUs) over a 10-year period.

## Methods

### Ethical approval and study design

This study was approved by the ethics committee at the Hamburg State Chamber of Physicians (registration no.: WF 012/13). The need for an informed consent was waived by the ethics committee, because data were retrieved from electronic health records. Critically ill patients with blood cultures positive for *Candida *spp. between October 2008 and July 2017 were included in the current analysis.

### Setting

The University Medical Center Hamburg-Eppendorf is a tertiary care hospital with 1738 hospital beds that treated 98,356 in-patients in 2017. The number of critical care patients increased over time, from 5999 patients in 2008 to 8817 patients in 2017. The Department of Intensive Care Medicine includes 12 multidisciplinary ICUs with a total of 140 ICU beds. The Department of Intensive Care Medicine has units for the postoperative care following cardiac, neurosurgery, traumatologic, vascular or visceral surgery. Our department routinely treats patients following solid organ or allogeneic stem cell transplantations. Mixed patient populations, both medical and surgical, are being treated on each unit.

### Data collection

The following demographic and clinical variables were collected from the electronic patient data management system (PDMS, ICM, Dräger, Lübeck, Germany): age, sex, body mass index, admission diagnosis, comorbidities, *Candida *spp., antifungal treatment; Simplified Acute Physiology Score II (SAPS II) and Sepsis-related Organ Failure Assessment (SOFA) score at time of culture positivity; septic shock; laboratory findings; CVC duration, number of CVCs per patient; length of ICU, and hospital stay.

In addition, the Candida score was calculated in all patients at the onset of clinical symptoms. The Candida score is a four-item clinical score and includes the components “severe sepsis”, “total parenteral nutrition”, “surgery”, and “multifocal Candida colonization” [15]. One point is added for each component resulting in a maximum score of 4. A score ≥ 3 is considered an accurate discriminative value to select patients with *Candida* colonization requiring antifungal treatment [16, 17].

Survival data at 28 and 180 days after culture positivity were obtained from medical records or from telephone follow-up with the patient, next-of-kin or caregiver.

### Microbiological assays

Aerobe and anaerobe blood cultures (BD Bactec Plus, BD, Heidelberg, Germany) were incubated in a Bactec instrument for up to 5 days. Bottles flagged positive were subjected to Gram-staining. Bottles showing growth of yeast and those that did not show visible microorganism were streaked onto Columbia blood and Sabouraud agar plates (Oxoid, Basingstoke, UK). After incubation at 37 °C for up to 48 h yeast were identified to the species level using whole cell mass spectrometry fingerprinting (Biotyper, Bruker Daltonics, Bremen, Germany), microscopy, or biochemical profiling (Auxacolor, Bio-Rad, Munich, Germany). All yeast isolates were subject to susceptibility testing using gradient strips. Susceptibility was interpreted according to the guidelines of the European Committee on Antimicrobial Susceptibility Testing (EUCAST).

### Antifungal management

We categorized antifungal treatment into (a) empirical treatment; (b) treatment upon confirmation of BSI with *Candida *spp.; (c) antifungal medication resistant to the culprit *Candida *spp.; (d) delayed antifungal treatment (> 24 h after culture positivity), and no antifungal treatment. Empirical antifungal treatment was administered to patients with risk factors for candidiasis and persistent fever despite antibacterial medication and/or a positive culture from non-sterile sites [12].

The initial treatment was considered adequate, if: (1) antifungal medication was administered empirically or within the first 24 h of culture positivity; (2) the isolated yeast was susceptible to the antifungal agent, and (3) source control, defined as CVC removal, was initiated within the first 48 h after blood culture positivity [18].

### Statistical analysis

Continuous variables are expressed as median with 1st to 3rd quantile. Categorical variables are given as absolute and relative numbers. The distribution of data was visually interpreted using histograms. Variables were compared between groups (survivors vs. non-survivors and *albicans* vs. non-*albicans*) with the Wilcoxon–Mann–Whitney U test, the Chi-square test or the Fisher’s exact test as appropriate.

A multivariable Cox proportional hazards regression model was performed to identify variables associated with mortality at 28 days and 180 days. One episode of candidemia per patient was analyzed. Variables that were considered clinically relevant or that had been identified as predictors of mortality in the EUCANDICU trial were included as covariates [1]: age, SOFA score, septic shock, *Candida *spp. (*albicans* vs. non-*albicans*), Candida score, admission diagnosis (medical, abdominal surgery, surgery other than abdominal), liver cirrhosis, immunosuppression (including solid organ or stem cell transplantation, acquired immune deficiency syndrome, immunosuppressive medication), mean CVC duration at culture positivity, mechanical ventilation, length of ICU stay at culture positivity, antifungal treatment (adequate, non-adequate, none), echinocandin. The proportional hazards assumption and the linearity assumption for continuous variables in the Cox model were assessed based on Schoenfeld and marginal residuals.

Cochran–Armitage trend test was performed to assess a change in distribution patterns of *Candida *spp. during the study period. All given *p*-values are of descriptive nature and not adjusted for multiple testing. Statistical analyses were performed using IBM® SPSS® Statistics 21 and R Version 3.5.1.

## Results

### Patient characteristics

**Fig. 1:**
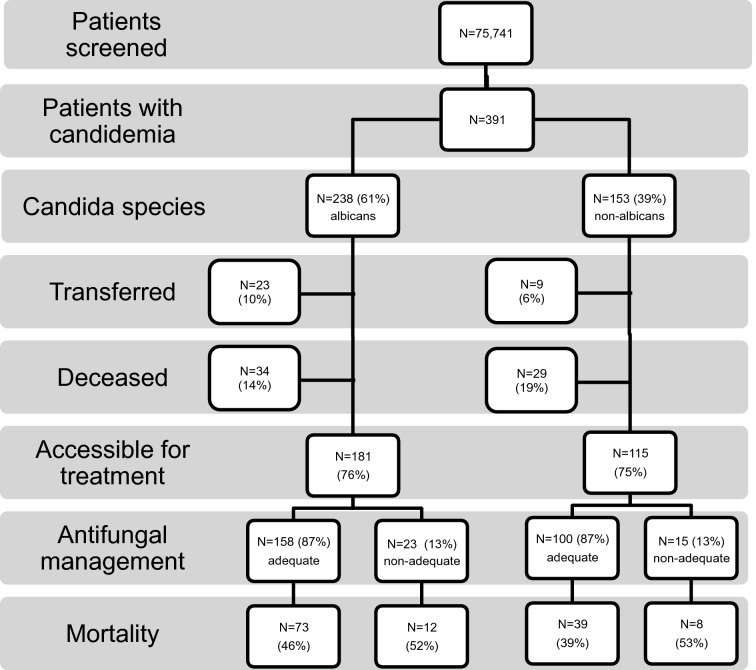
Flow of participants throughout the study. “Accessible for antifungal treatment” refers to patients, who were not transferred or died prior to diagnosis of bloodstream infection caused by *Candida *spp

For this retrospective study, we identified 391 patients with *Candida* BSI, who were treated in one of 12 ICUs. The first patient was enrolled on October 24th, 2008 and the last 180-day follow-up was on January 13 th, 2018. During the study period from January 2008 until December 2017, 75.741 patients were admitted to our department with a crude mortality of 8.9%. *Candida* BSI was found in 391 patients (0.5%), accounting for an incidence of 4.8/1000 ICU admissions. For the flow of participants throughout the study, see Fig. 1.

Table 1 shows baseline demographic characteristics, comorbid conditions, admission diagnoses, and treatment-related variables for survivors and non-survivors at 180 days. The median age was 65.2 years (IQR: 56.0–73.7); 61.4% (*n* = 240) patients were male. There were no significant differences in demographic characteristics between survivors and non-survivors. Patients who died had higher SAPS II, SOFA, and Candida scores, increased length of hospital and ICU stays. Non-survivors suffered more frequently from liver cirrhosis and septic shock, required extracorporeal organ support before and after *Candida* BSI more frequently, had higher mean CVC duration, and increased length of ICU and hospital stays. Selected patient characteristics throughout the study period are presented in Additional file 1. A list of referring specialties is provided in Additional file 2.

**Table 1:** Baseline demographic and clinical characteristics, admission diagnosis, treatment-related variables and microbiological findings in critically ill patients with laboratory-confirmed bloodstream infection with *Candida *spp. Continuous variables are given as median (1st to 3rd quantile), categorical variables are given as *n* (%)

	Study participants *n* = 391	Non-survivors *n* = 234	Survivors *n* = 157	Descriptive *p*-value (unadjusted)^a^
Demographics				
Age (years)	65 (56–74)	65 (57–73)	65 (53–74)	0.614
Male gender	240 (61)	151 (65)	89 (57)	0.138
Comorbid conditions on admission				
Coronary heart disease	100 (26)	63 (27)	37 (24)	0.480
Diabetes mellitus	99 (25)	62 (27)	37 (24)	0.554
COPD	57 (15)	31 (13)	26 (17)	0.383
Liver cirrhosis	70 (18)	56 (24)	14 (9)	< 0.001
Chronic kidney disease	205 (52)	129 (55)	76 (48)	0.216
Hematologic malignancy	28 (7)	22 (9)	6 (4)	0.045
Solid tumor	139 (36)	82 (35)	57 (36)	0.830
Implantable cardiac devices	32 (22)	21 (23)	11 (20)	0.837
Stem cell transplantation	5 (1)	4 (2)	1 (1)	0.355
Human immunodeficiency virus	0 (0)	2 (1)	2 (1)	0.245
Solid organ transplantation	22 (6)	13 (6)	9 (6)	0.941
Admission diagnosis				0.041
Medical	175 (45)	116 (50)	59 (38)	
Abdominal surgery	133 (34)	76 (33)	57 (36)	
Surgery other than abdominal	83 (21)	42 (18)	41 (26)	
ICU stay				
Arterial catheter	363 (93)	216 (92)	147 (94)	0.619
Mechanical ventilation	362 (93)	218 (93)	144 (92)	0.594
Red blood cell transfusion	2 (0–7)	3 (0–9)	2 (0–6)	0.056
SAPS II score^b^	44 (35–53)	48 (40–57)	36 (31–47)	< 0.001
SOFA score^b^	8 (5–11)	10 (7–12)	6 (3–9)	< 0.001
Candida score^b^	3 (2–4)	3 (2–4)	2 (1–4)	< 0.001
Sepsis	287 (73)	198 (85)	89 (57)	< 0.001
Septic shock	151 (39)	128 (55)	23 (15)	< 0.001
Extracorporeal organ support before culture positivity	182 (47)	129 (55)	53 (34)	< 0.001
After culture positivity	210 (54)	156 (68)	54 (34)	< 0.001
ICU length of stay (days)	28 (15–51)	25 (13–48)	33 (21–54)	0.003
Before culture positivity	12 (5–23)	12 (5–23)	13 (5–23)	0.638
Hospital length of stay (days)	37 (22–66)	30 (16–58)	50 (33–72)	< 0.001
Before culture positivity	15 (8–30)	16 (8–31)	14 (8–29)	0.428
Inflammatory parameters at culture positivity				
White blood cell count (10^9^/l)	13 (9–20)	14 (2–22)	12 (9–16)	0.005
Procalcitonin (µg/l)	2 (1–7)	3 (1–7)	2 (1–8)	0.042
C-reactive protein (mg/dl)	124 (71–189)	129 (71–193)	121 (68–187)	0.682
Lactate (mmol/l)	2 (1–4)	3 (2- 5)	2(1–2)	< 0.001
Central venous catheter (CVC)				
Number of CVCs per patient	5 (3–8)	5 (3–8)	5 (3–9)	0.232
CVC overall time in place (hours)	726 (326–1432)	698 (306–1400)	772 (344–1443)	0.710
Max. time in place per CVC (hours)	242 (162–308)	230 (156–308)	257 (188–308)	0.204
Min. time in place per CVC (hours)	43 (15–87)	38 (12–68)	61 (18–99)	0.001
Mean time in place per CVC (hours)	141 (101–176)	135(94–168)	157 (109–190)	0.009
Time in place per CVC before first Candida finding (hours)	232(19–563)	237 (30–627)	223 (6–535)	0.444
Microbiological findings				
Candida albicans	238 (61)	135 (58)	103 (66)	0.116
Candida non-albicans species	153 (39)	99 (42)	54 (34)	
Gram-positive bacteremia	155 (40)	85 (37)	70 (45)	0.109
Gram-negative bacteremia	38 (10)	19 (8)	19 (12)	0.193

^a^Wilcoxon–Mann–Whitney U test was performed for continuous variables and Fisher’s exact test for categorical variables. ^b^Scores at culture positivity. COPD: chronic obstructive pulmonary disease. SAPS II: Simplified Acute Physiology Score II. ICU: intensive care unit. SOFA score: Sepsis-related organ failure assessment score

### Candida species

**Fig. 2:**
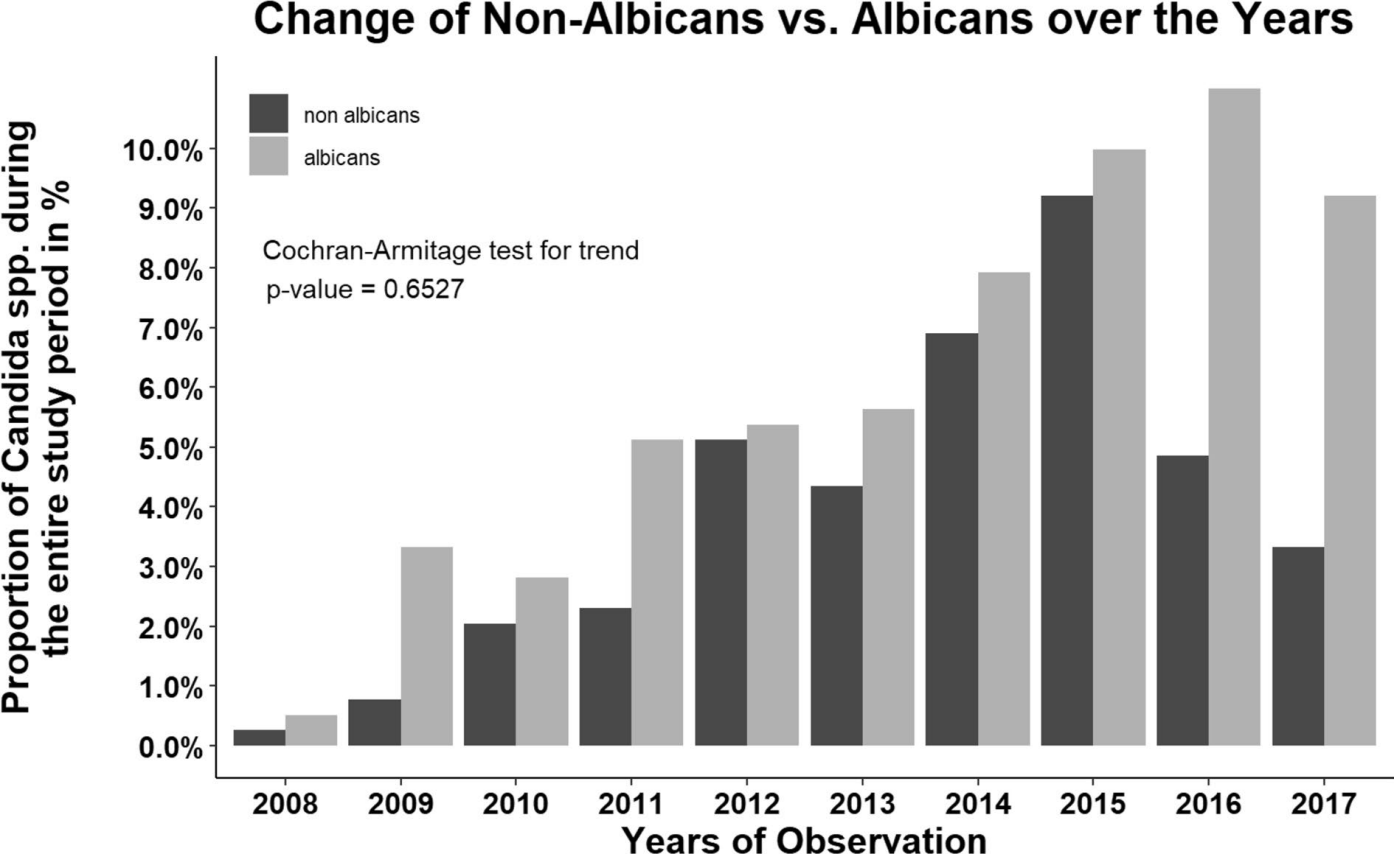
Distribution of *Candida albicans* versus non-*albicans Candida *spp. between 2008 and 2017

Microbiological assays identified ten different *Candida *spp. in our study population (Additional file 3). The most frequent species was *C. albicans* (*n* = 238, 60.9%), followed by *C. glabrata* (*n* = 76, 19.4%), *C. parapsilosis* (*n* = 26, 6.6%) and *C. tropicalis* (*n* = 23, 5.9%). *C. dubliniensis* was identified in 13 patients (3.3%) and *C. krusei* in 7 patients (1.8%). *C. lusitaniae* and *C. kefyr* were detected in 3 patients (0.8%) each. Only one patient (0.3%) each was tested positive for *C. norvegensis* or *C. guilliermondii*. *C. auris* was not identified in any blood culture. Figure 2 shows the distribution of non-*albicans* and *C. albicans* throughout the study period from 2008 until 2017. The *albicans*/non-*albicans* ratio did not change over this time period (*p* = 0.653). For details on species distribution, see Additional file 3.

Of note, the number of *Candida albicans* and non-*albicans Candida *spp. were similarly distributed between 2012 and 2015 (2012; 48.8%, 2013; 43.6%, 2014; 46.6%, 2015 48.0% non-*albicans Candida *spp*.*). By contrast, the proportion of non-*albicans Candida *spp. decreased in 2016 and 2017 (30.6% and 26.5%, respectively, Additional file 4).

### Antifungal management

**Fig. 3:**
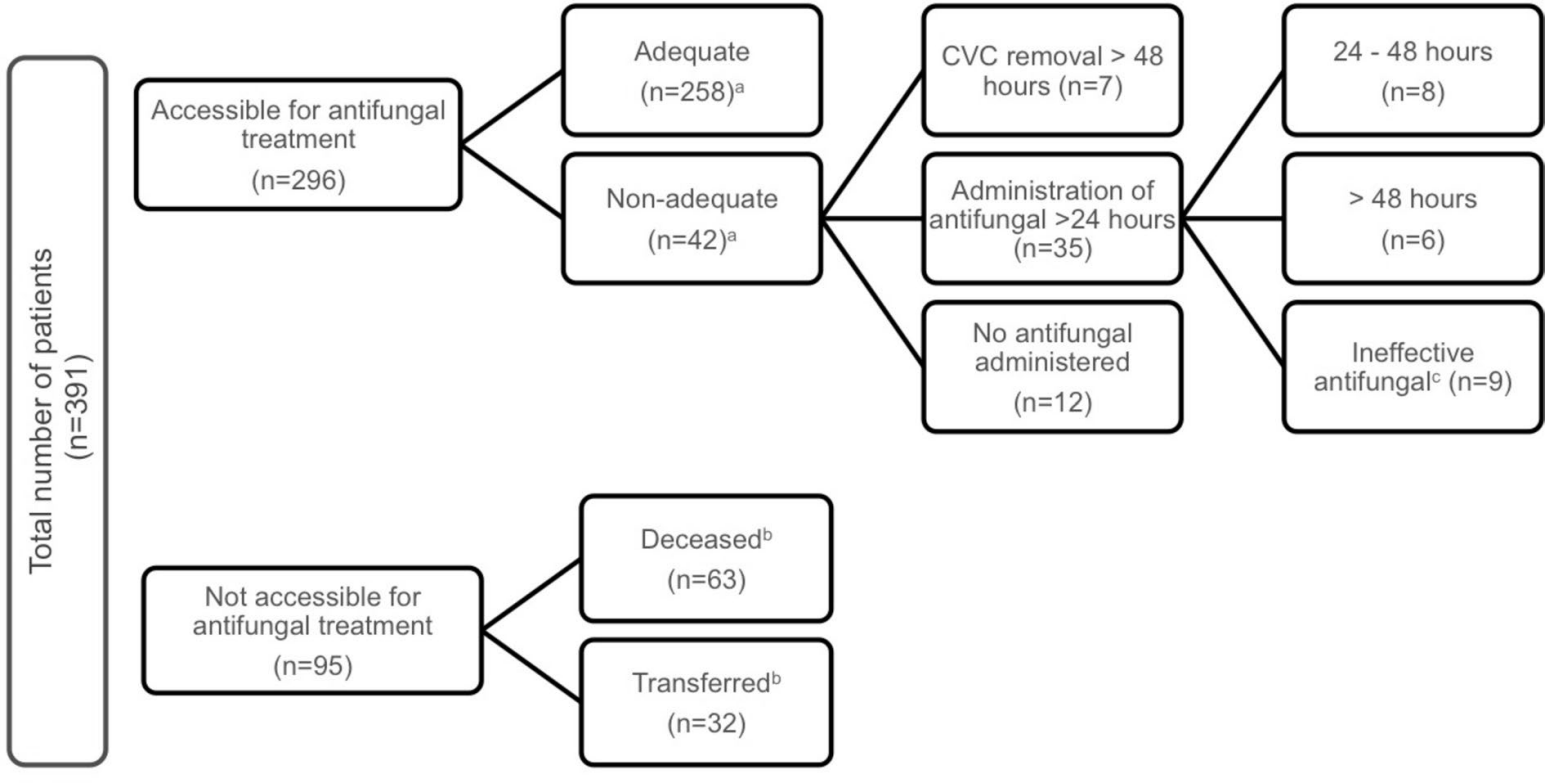
Antifungal management in 391 critically ill patients with *Candida*-induced bloodstream infection. ^a^Numbers do not add up to 296 because more than one criterion was found in some patients. ^b^Refers to patients, who died or were transferred to another hospital/rehabilitation facility, before microbiological results were obtained. ^c^*Candida *spp. was not susceptible to antifungal substance administered

Two hundred ninety-six patients (75.7%) were accessible for antifungal treatment. In the remaining patients, 63 (16.1%) died and 32 (8.2%) had been transferred before microbiological results were obtained. Empirical antifungal medication was administered in 88/391 patients (22.5%), Table 2. The criteria for adequate antifungal treatment were fulfilled by 258/296 (87.5%) patients of those accessible for treatment (Fig. 3). Treatment was considered non-adequate in 7/296 (2.4%) patients for CVC removal more than 48 h after culture positivity with *Candida *spp. In another 35/296 (11.8%) patients, treatment was delayed or not susceptible for the BSI-causing *Candida *spp. (Fig. 3). The three most frequently used substances were fluconazole, anidulafungin, and caspofungin. For details on antifungal medication, see Table 2.

**Table 2:** Antifungal treatment in critically ill patients with laboratory-confirmed bloodstream infection with *Candida *spp. Continuous variables are given as median (1st to 3rd quantile), categorical variables are given as *n* (%)

	Study participants *n* = 391	Non-survivors *n* = 234	Survivors *n* = 157	Descriptive p-value (unadjusted)^a^
Antifungal strategy				0.128
Targeted ^b^	174 (44.5%)	96 (41.0%)	78 (49.7%)	
Empirical ^c^	88 (22.5%)	53 (22.6%)	35 (22.3%)	
Resistant ^d^	8 (2.0%)	3 (1.3%)	5 (3.2%)	
Delayed ^e^	14 (3.6%)	8 (3.4%)	6 (3.8%)	
None	107 (27.4%)	74 (31.6%)	33 (21.0%)	
Treatment with echinocandin	187 (47.8%)	108 (46.2%)	79 (50.3%)	0.419
Empirical therapy ^c^				0.435
Echinocandin	41 (10.5%)	24 (10.3%)	17 (10.8%)	
Azoles	48 (12.3%)	32 (13.7%)	16 (10.2%)	
Amphotericin B	6 (1.5%)	2 (0.9%)	4 (2.5%)	
Duration of antifungal treatment (days)	8 (0–17)	5 (0–15)	11 (3–19)	< 0.001
Antifungals ^f^				
Fluconazole	159 (40.7)	83 (35.5)	76 (48.4)	
Anidulafungin	129 (33.0)	75 (32.1)	54 (34.4)	
Caspofungin	66 (16.9)	37 (15.8)	29 (18.5)	
Voriconazole	22 (5.6)	18 (7.7)	4 (2.5)	
Amphotericin B	16 (4.1)	8 (3.4)	8 (5.1)	
Posaconazole	1 (0.3)	1 (0.4)	0 (0)	
Itraconazole	1 (0.3)	1 (0.4)	0 (0)	

^a^Wilcoxon–Mann–Whitney U test was performed for continuous variables and Fisher’s exact test for categorical variables. ^b^Targeted treatment was initiated within 24 h of culture positivity. ^c^Empirical treatment was administered in patients with risk factors for candidemia and persistent fever despite antibacterial medication or positive culture from non-sterile sites. Empirical treatment refers to antifungal medication that was effective according to antifungal susceptibility testing. ^d^Resistant: antifungal treatment that was not effective against the detected *Candida* species. ^e^Delayed: antifungal treatment was started more than 24 h after culture positivity. ^f^Antifungal substances used throughout the study period between 2008 and 2017. The total number does not add up to 284 because of escalation/de-escalation of antifungal treatment

### Follow-up 28 and 180 days after Candida BSI

The mortality after 28 days was 47.3% (*n* = 185) and increased to 59.8% (*n* = 234) after 180 days, counted from the first day of BSI with *Candida *spp. Higher age, SOFA and Candida scores, liver cirrhosis, septic shock, CVC duration, and length of ICU stay were risk factors for mortality at 28 and 180 days in the multivariable Cox regression. Patients, who received adequate and non-adequate antifungal treatment for *Candida* BSI, and patients undergoing abdominal surgery had a higher probability of survival at 28 and 180 days. Echinocandins for antifungal treatment were associated with survival at 28, but not at 180 days. Details on the multivariable models are presented in Table 3 and Additional file 5.

**Table 3:** Probability of death 28 and 180 days after blood cultures were tested positive for *Candida *spp. 381 observations, 178 events (28 days), and 227 events (180 days) were included in the multivariable analysis

Probability of death	At 28 days		At 180 days	
Variable	Hazard ratio	95% CI	Hazard ratio	95% CI
Age (per year increase)	1.02	1.00–1.03	1.02	1.01–1.03
Liver cirrhosis (vs. none)	1.61	1.09–2.38	1.54	1.07–2.20
Immunosuppression^a^ (vs. none)	0.97	0.71–1.32	0.84	0.63–1.11
SOFA score (per point increase)	1.14	1.08–1.19	1.12	1.07–1.17
Septic shock	2.60	1.77–3.82	2.41	1.73–3.37
Admission diagnosis				
Abdominal surgery vs. medical	0.55	0.38–0.81	0.66	0.48–0.91
Other surgery vs. medical	0.79	0.52–1.20	0.70	0.48–1.03
Abdominal surgery vs. other surgery	1.43	0.89–2.30	1.06	0.71–1.60
Days at ICU before Candida BSI (per day increase)	1.01	1.00–1.01	1.01	1.00—1.01
Mechanical ventilation (vs. none)	0.95	0.49–1.82	0.77	0.44–1.34
Extracorporeal organ support before Candida BSI (vs. none)	1.23	0.85–1.78	1.28	0.93–1.76
Mean CVC duration (per h increase)	1.00	1.00–1.00	1.00	1.00–1.00
Candida score (per point increase)	1.21	1.06–1.37	1.25	1.11–1.40
*Candida* species (albicans vs. non-albicans)	1.14	0.82–1.59	1.08	0.80–1.44
Treatment with echinocandins (vs. none or other antifungals)	0.67	0.45–0.99	0.79	0.56–1.11
Antifungal treatment:				
Non-adequate vs. none	0.23	0.10–0.56	0.31	0.16—0.62
Adequate vs. none	0.30	0.20–0.45	0.36	0.24–0.52
Adequate vs. non-adequate	0.78	0.34–1.81	0.88	0.47–1.65

SAPS II: Simplified Acute Physiology Score II at time of culture positivity. ICU: intensive care unit. BSI: bloodstream infection. CVC: central venous catheter. SOFA score: Sepsis-related organ failure assessment score. ^a^Immunosuppression: stem cell transplantation, acquired immunodeficiency syndrome, solid organ transplantation, immunosuppressive medication. The criteria for adequate antifungal treatment were fulfilled, if (1) antifungal medication was administered within the first 24 h of culture positivity, (2) substance dosage was weight adjusted and in accordance with current recommendations, (3) the isolated yeast was susceptible to the antifungal agent, and (4) source control was initiated within the first 48 h after blood culture positivity

## Discussion

In this single-center retrospective study, including 391 critically ill patients with *Candida* BSI, we observed a high mortality 28 and 180 days after culture positivity. We identified higher age, a history of liver cirrhosis, high SOFA and Candida scores, increased length of ICU stay, and the presence of septic shock as risk factors for mortality after *Candida* BSI. Patients, who had abdominal surgery, and patients receiving any empirical antifungal agent had a reduced risk of mortality compared to medical admission type and no antifungal treatment, respectively. Throughout the study period of 10 years, we did not observe a significant change in the distribution pattern of yeast species and no multidrug-resistant *Candida auris *spp*.* was identified.

We found a mortality rate of 47% 28 days after diagnosis of *Candida* BSI. Our results are in line with findings from previous studies. In one of the largest European multicenter trials on the epidemiology of ICU-acquired candidiasis, the 30-day mortality rate was 42% [1]. Similarly, a 30-day-mortality of 45% has been reported after candidemia in internal medicine wards [19]. The French Mycoses study group observed 50% survival in a mixed ICU population 30 days after candidemia [11], which is similar to the findings of a retrospective observation by Ghanem-Zoubi and colleagues [3]. An analysis from prospectively collected data in the French REA-RAISIN network showed a mortality rate of 52% among patients with ICU-acquired candidemia in 213 ICUs [20].

We found that adequate antifungal management, defined by the start of antifungal medication, susceptibility of *Candida *spp., and time point of CVC removal, was a predictor of survival after 28 and 180 days. Interestingly, multivariable analysis showed that also non-adequate antifungal treatment including delayed administration of antifungal medication or resistant drugs was associated with an increased probability of survival. By contrast, adequate antifungal treatment, does not seem to be superior with regard to survival after 28 or 180 days when compared to non-adequate antifungal management. Several factors may be accountable for the non-superiority of adequate antifungal treatment over non-adequate antifungal management with regard to survival in our study population. First, there are no detailed uniform criteria for adequate antifungal treatment. We used a rather strict definition that has been proposed previously [18]. Following a more liberal definition, 21 (7.1%) more patients would have fulfilled the criteria for adequate treatment, which may have altered the results of our analysis.

The influence of different definitions of appropriate antifungal treatment on the strength of association with mortality after candidemia has been reported by another retrospective trial. Three definitions of appropriate empiric treatment and their association with 30-day mortality were compared in a cohort of 302 critically ill patients [3]. Interestingly, statistical associations varied by definition, which underlines the impact of treatment criteria on outcome prediction.

Second, the susceptibility of *Candida *spp. to specific antifungal drugs may not necessarily correlate with outcome. Ghrenassia and colleagues observed that *Candida* susceptibility was not associated with survival after candidemia in immunocompromised patients [21]. Neither was the choice of antifungal agent in favor of echinocandins. As opposed to the treatment regimen in our study, antifungal prophylaxis was used by Ghenrassia et al. which may limit the comparability with our results.

Third, the fact that any empiric antifungal treatment was initiated, irrespective of susceptibility and time point, may mirror awareness and disease recognition of *Candida* BSI by the ICU team, which might explain the positive association with survival at 28 and 180 days.

We found an association between treatment with echinocandins and survival after 28 days. By contrast, mortality after 180 days was not influenced by echinocandins for antifungal management. There remains controversy on the impact of echinocandins on mortality [22–25]. Of note, other factors such as comorbid conditions, disease severity, and appropriate antifungal management including source control may be more relevant for survival after candidemia, which may explain the conflicting results [22]. We found a beneficial effect of echinocandins on short-term mortality after 28 days that did not sustain the 180-day follow-up. Long-term survival in critically ill patients may be determined by the underlying condition and disease severity rather than the type of initial antifungal medication.

Compared with medical patients, patients undergoing abdominal surgery had a lower risk of mortality at 28 and 180 days. Similar results have been reported from the Spanish CANDIPOP trial that found a lower mortality from candidemia in surgical wards compared with medical wards [26]. Better outcome in surgical patients might be attributable to lower disease severity, fewer immunosuppression, and organ failure [26].

Preexisting liver cirrhosis was associated with increased 28- and 180-day mortality in our study population, which confirms the results of previous studies [27]. Next to the SOFA score as an indicator of morbidity in critical care patients and the presence of septic shock, we found an association between the Candida score and reduced survival at 28 and 180 days [28]. All factors that were identified as predictors of poor outcome in our study population have been linked with increased mortality in ICU patients before [29–31]. Thus, our results confirm the importance of disease severity and multiorgan dysfunction for the prognosis after *Candida* BSI.

*Candida albicans* was the most frequently encountered yeast isolate in our study population, followed by *Candida glabrata* and *Candida parapsilosis*. Our findings are in line with data from the multicenter EUCANDICU trial reporting similar distribution patterns. There are conflicting results on a shift in *Candida *spp. towards non-*albicans* isolates. Several Italian studies report a decrease of *Candida albicans* and an increase of non-*albicans Candida *spp*. *[32, 33]. By contrast, two observational studies in French and Belgian ICUs did not observe a species shift and describe patterns similar to our results with a predominance of *Candida albicans* [34, 35]. We found a relatively stable *Candida albicans*/non-*albicans* ratio between 2012 and 2015. Interestingly, there was a substantial shift towards *Candida albicans* in the years 2016 and 2017. The decrease of non-*albicans* species in favor of *Candida albicans* might be attributable to episodic fluctuations that have been observed periodically [35]. Of note, *Candida auris*, a multidrug-resistant pathogen, which was first described in Japan in 2009 [36], has been recently associated with outbreaks worldwide, including Europe, could not be detected in our study population [37–40].

Our study has several limitations that need to be addressed. Results from this retrospective analysis are of exploratory nature and should be interpreted with caution. Another limitation of this study is the lack of biomarkers that have since been used to diagnose and monitor therapy [41, 42]. However, these biomarkers ((1,3) beta-D glucan and Candida-Ag) were not widely available in our retrospective cohort and have only become established in recent years.

We did not analyze total parenteral nutrition, which is one of the leading risk factors for increased mortality after *Candida* BSI, as a single variable, since it is included in the Candida score. Numerous parameters have been identified as risk factors for mortality after candidemia in critically ill patients. These include a history of congestive heart failure, the presence of solid tumors with metastases, red blood cell transfusions, and the duration of antifungal treatment [24, 43–45]. For survival analysis, we selected covariates that were considered clinically relevant or factors that had been associated with 30-day mortality in a multinational observational study in 23 European ICUs [1]. Other covariates and unknown variables that were omitted from our analyses may have biased the results of this study, which has to be considered when interpreting our findings.

Source control was defined as CVC removal in our study. We did not include other management strategies such as the removal of implanted devices, drainage of infected fluid collections or surgical debridement of infected solid tissue. We chose this definition, because we aimed to analyze the impact of measures that were immediately performed by the ICU team rather than surgical procedures.

One strength of our study is the long observation period of 10 years that allows the analysis of species distribution independently from seasonal fluctuations. Importantly, we obtained microbiological results from a mixed population of critically ill patients, including surgical and medical conditions, hematologic malignancy, solid and organ stem cell transplantation.

Most previous trials that assessed outcome after candidemia report short-term mortality rates after one month [1, 46], since the 30-day outcome is traditionally used as an endpoint in critical care trials [47]. We aimed to evaluate outcome beyond this time period and assessed long-term survival 180 days after diagnosis of *Candida* BSI in addition to 28-day mortality.

## Conclusion

*Candida* BSI is still a serious and life-threatening disease with very high morbidity and mortality in critically ill patients. Our study demonstrates that prompt antifungal treatment improves the likelihood of survival, regardless of yeast susceptibility and exact time point of administration. Patients with liver cirrhosis and high disease severity as expressed by the SAPS II and the Candida scores, and organ failure requiring extracorporeal support are at increased risk of death 28 and 180 days after culture positivity, whereas surgical patients seem to have more favorable outcome after *Candida* BSI. Species distribution remained relatively constant throughout a 10-year period with *Candida albicans* as the predominant yeast species. Our results underline the importance of rapid treatment of *Candida* BSI and point to the need for a uniform definition of adequate antifungal management.

## Supplementary information

**Additional file 1.** a-d: Selected patient characteristics throughout the study period between 2008 and 2017. a) Age, b) Sepsis-related Organ Failure Assessment (SOFA) score, and c) Simplified Acute Physiology Score on the day of *Candida* BSI are given as median. d) Surgical admission in %.

**Additional file 2.** Referring/co-managing specialties divided by mortality after 180 days. Data are given as absolute and relative numbers. ^a^Fisher’s exact test was performed for categorical variables.

**Additional file 3.** Distribution of Candida spp. in survivors (*n* = 157) and non-survivors (*n* = 234) at 180 days. BSI: bloodstream infection.

**Additional file 4.** Distribution of *C. albicans* versus non-*albicans Candida *spp. between 2008 and 2017.

**Additional file 5.** Predictors of mortality 180 days after blood cultures positive for *Candida *spp. were obtained. Squared symbols indicate hazard ratios, error bars indicate 95% confidence intervals.

## Data Availability

The datasets generated and/or analyzed during the current study are not publicly available due to data protection of the patients, but are available from the corresponding author on reasonable request.

## Abbreviations

BSI: Bloodstream Infections
CVC: Central venous catheter
ICU: Intensive care unit
SAPS II: Simplified Acute Physiology Score II
SOFA: Sepsis-related Organ Failure Assessment

## References

[1] Bassetti M, Giacobbe DR, Vena A, Trucchi C, Ansaldi F, Antonelli M (2019). Incidence and outcome of invasive candidiasis in intensive care units (ICUs) in Europe: results of the EUCANDICU project. Crit Care.

[2] Tsay SV, Mu Y, Williams S, Epson E, Nadle J, Bamberg WM (2020). Burden of candidemia in the united states, 2017. Clin Infect Dis..

[3] Ghanem-Zoubi N, Zorbavel D, Khoury J, Geffen Y, Qasum M, Predescu S (2018). The association between treatment appropriateness according to EUCAST and CLSI breakpoints and mortality among patients with candidemia: a retrospective observational study. Eur J Clin Microbiol Infect Dis.

[4] Luzzati R, Merelli M, Ansaldi F, Rosin C, Azzini A, Cavinato S (2016). Nosocomial candidemia in patients admitted to medicine wards compared to other wards: a multicentre study. Infection.

[5] Schwab F, Geffers C, Behnke M, Gastmeier P (2018). ICU mortality following ICU-acquired primary bloodstream infections according to the type of pathogen: a prospective cohort study in 937 Germany ICUs (2006–2015). PLoS ONE.

[6] Novosad SA, Fike L, Dudeck MA, Allen-Bridson K, Edwards JR, Edens C (2020). Pathogens causing central-line-associated bloodstream infections in acute-care hospitals-United States, 2011–2017. Infect Control Hosp Epidemiol..

[7] Kett DH, Azoulay E, Echeverria PM, Vincent JL (2011). Extended Prevalence of infection in ICUSGOI. Candida bloodstream infections in intensive care units: analysis of the extended prevalence of infection in intensive care unit study. Crit Care Med..

[8] Montagna MT, Caggiano G, Lovero G, De Giglio O, Coretti C, Cuna T (2013). Epidemiology of invasive fungal infections in the intensive care unit: results of a multicenter Italian survey (AURORA Project). Infection..

[9] Pfaller M, Neofytos D, Diekema D, Azie N, Meier-Kriesche HU, Quan SP (2012). Epidemiology and outcomes of candidemia in 3648 patients: data from the Prospective Antifungal Therapy (PATH Alliance(R)) registry, 2004–2008. Diagn Microbiol Infect Dis.

[10] Garnacho-Montero J, Diaz-Martin A, Garcia-Cabrera E, de Pipaon RPM, Hernandez-Caballero C, Aznar-Martin J (2010). Risk factors for fluconazole-resistant candidemia. Antimicrob Agents Chemotherapy..

[11] Lortholary O, Desnos-Ollivier M, Sitbon K, Fontanet A, Bretagne S, Dromer F (2011). Recent exposure to caspofungin or fluconazole influences the epidemiology of candidemia: a prospective multicenter study involving 2441 patients. Antimicrob Agents Chemotherapy..

[12] Pappas PG, Kauffman CA, Andes DR, Clancy CJ, Marr KA, Ostrosky-Zeichner L, Guideline for the Management of Candidiasis, CP (2016). Update by the Infectious Diseases Society of America. Vol. 62, Clinical infectious diseases: an official publication of the Infectious Diseases Society of. America.

[13] Bassetti M, Merelli M, Ansaldi F, de Florentiis D, Sartor A, Scarparo C, et al. Clinical and therapeutic aspects of candidemia: a five year single centre study. Consolaro MEL, editor. PLoS ONE. 2015;10(5):e0127534. 10.1371/journal.pone.0127534 PMC4444310 26010361

[14] Garey KW, Rege M, Pai MP, Mingo DE, Suda KJ, Turpin RS (2006). Time to initiation of fluconazole therapy impacts mortality in patients with candidemia: a multi-institutional study. Clin Infect Dis.

[15] León C, Ruiz-Santana S, Saavedra P, Almirante B, Nolla-Salas J, Alvarez-Lerma F (2006). A bedside scoring system (“Candida score”) for early antifungal treatment in nonneutropenic critically ill patients with Candida colonization. Crit Care Med.

[16] Bassetti M, Garnacho-Montero J, Calandra T, Kullberg B, Dimopoulos G, Azoulay E (2017). Intensive care medicine research agenda on invasive fungal infection in critically ill patients. Intensive Care Med..

[17] León C, Ruiz-Santana S, Saavedra P, Galván B, Blanco A, Castro C (2009). Usefulness of the “Candida score” for discriminating between Candida colonization and invasive candidiasis in non-neutropenic critically ill patients: a prospective multicenter study. Crit Care Med.

[18] Bassetti M, Righi E, Ansaldi F, Merelli M, Trucchi C, Cecilia T (2014). A multicenter study of septic shock due to candidemia: outcomes and predictors of mortality. Intensive Care Med..

[19] Sbrana F, Sozio E, Bassetti M, Ripoli A, Pieralli F, Azzini AM (2018). Independent risk factors for mortality in critically ill patients with candidemia on Italian Internal Medicine Wards. Intern Emerg Med..

[20] Baldesi O, Bailly S, Ruckly S, Lepape A, L'Heriteau F, Aupee M (2017). ICU-acquired candidaemia in France: epidemiology and temporal trends, 2004–2013—a study from the REA-RAISIN network. J Infect.

[21] Ghrenassia E, Mokart D, Mayaux J, Demoule A, Rezine I, Kerhuel L (2019). Candidemia in critically ill immunocompromised patients: report of a retrospective multicenter cohort study. Ann Intensive Care.

[22] Bennett JE, Powers JH (2018). Candidemia in the ICU: does initial antifungal matter?. Crit Care Med.

[23] Falcone M, Tiseo G, Gutiérrez-Gutiérrez B, Raponi G, Carfagna P, Rosin C (2019). Impact of initial antifungal therapy on the outcome of patients with Candidemia and septic shock admitted to medical wards: a propensity score-adjusted analysis. Open Forum Infect Dis..

[24] Garnacho-Montero J, Diaz-Martin A, Cantón-Bulnes L, Ramírez P, Sierra R, Arias-Verdú D (2018). Initial antifungal strategy reduces mortality in critically Ill patients with candidemia: a propensity score-adjusted analysis of a multicenter study. Crit Care Med.

[25] Reboli AC, Rotstein C, Pappas PG, Chapman SW, Kett DH, Kumar D (2007). Anidulafungin versus fluconazole for invasive candidiasis. N Engl J Med..

[26] Vena A, Bouza E, Valerio M, Padilla B, Paño-Pardo JR, Fernández-Ruiz M, et al. Candidemia in non-ICU surgical wards: Comparison with medical wards. Sturtevant J, editor. PLoS ONE. 2017;12(10):e0185339. 10.1371/journal.pone.0185339 PMC5646772 29045423

[27] De Rosa FG, Trecarichi EM, Montrucchio C, Losito AR, Raviolo S, Posteraro B (2013). Mortality in patients with early- or late-onset candidaemia. J Antimicrob Chemother.

[28] Lambden S, Laterre PF, Levy MM, Francois B (2019). The SOFA score-development, utility and challenges of accurate assessment in clinical trials. Crit Care..

[29] Haltmeier T, Inaba K, Effron Z, Dollbaum R, Shulman IA, Benjamin E (2015). Candida score as a predictor of worse outcomes and mortality in severely injured trauma patients with positive candida cultures. Am Surg.

[30] Le Gall JR, Lemeshow S, Saulnier F (1993). A new simplified acute physiology score (SAPS II) based on a European/North American multicenter study. JAMA.

[31] McPhail MJW, Parrott F, Wendon JA, Harrison DA, Rowan KA, Bernal W (2018). Incidence and outcomes for patients with cirrhosis admitted to the united kingdom critical care units. Crit Care Med.

[32] Barchiesi F, Orsetti E, Gesuita R, Skrami E, Manso E, Candidemia Study G (2016). Epidemiology, clinical characteristics, and outcome of candidemia in a tertiary referral center in Italy from 2010 to 2014. Infection.

[33] Tortorano AM, Dho G, Prigitano A, Breda G, Grancini A, Emmi V (2012). Invasive fungal infections in the intensive care unit: a multicentre, prospective, observational study in Italy (2006–2008). Mycoses.

[34] Goemaere B, Becker P, Van Wijngaerden E, Maertens J, Spriet I, Hendrickx M (2018). Increasing candidaemia incidence from 2004 to 2015 with a shift in epidemiology in patients preexposed to antifungals. Mycoses.

[35] Sasso M, Roger C, Sasso M, Poujol H, Barbar S, Lefrant JY (2017). Changes in the distribution of colonising and infecting Candida spp. isolates, antifungal drug consumption and susceptibility in a French intensive care unit: a 10-year study. Mycoses..

[36] Satoh K, Makimura K, Hasumi Y, Nishiyama Y, Uchida K, Yamaguchi H (2009). Candida auris sp. Nov., a novel ascomycetous yeast isolated from the external ear canal of an inpatient in a Japanese hospital. Microbiol Immunol..

[37] Chowdhary A, Sharma C, Meis JF (2017). Candida auris: a rapidly emerging cause of hospital-acquired multidrug-resistant fungal infections globally. PLoS Pathog.

[38] Cortegiani A, Misseri G, Giarratano A, Bassetti M, Eyre D (2019). The global challenge of Candida auris in the intensive care unit. Crit Care.

[39] Eyre DW, Sheppard AE, Madder H, Moir I, Moroney R, Quan TP (2018). A Candida auris outbreak and its control in an intensive care setting. N Engl J Med.

[40] Schelenz S, Hagen F, Rhodes JL, Abdolrasouli A, Chowdhary A, Hall A (2016). First hospital outbreak of the globally emerging Candida auris in a European hospital. Antimicrob Resistance Infect Control.

[41] Martin-Loeches I, Antonelli M, Cuenca-Estrella M, Dimopoulos G, Einav S, De Waele JJ (2019). ESICM/ESCMID task force on practical management of invasive candidiasis in critically ill patients. Intensive Care Med.

[42] Rouze A, Loridant S, Poissy J, Dervaux B, Sendid B, Cornu M (2017). Biomarker-based strategy for early discontinuation of empirical antifungal treatment in critically ill patients: a randomized controlled trial. Intensive Care Med.

[43] Alves PGV, Melo SGO, de Bessa MAS, de Brito MO, de Menezes RP, de Araújo LB (2020). Risk factors associated with mortality among patients who had candidemia in a university hospital. Rev Soc Bras Med Trop..

[44] Marchena-Gomez J, Saez-Guzman T, Hemmersbach-Miller M, Conde-Martel A, Morales-Leon V, Bordes-Benitez A (2009). Candida isolation in patients hospitalized on a surgical ward: significance and mortality-related factors. World J Surg..

[45] Weinberger M, Leibovici L, Perez S, Samra Z, Ostfeld I, Levi I (2005). Characteristics of candidaemia with Candida-albicans compared with non-albicans Candida species and predictors of mortality. J Hosp Infect.

[46] Chakrabarti A, Sood P, Rudramurthy SM, Chen S, Kaur H, Capoor M (2015). Incidence, characteristics and outcome of ICU-acquired candidemia in India. Intensive Care Med..

[47] Groeneveld JA (2016). Mortality as an endpoint in studies in critically ill patients: a reappraisal of definitions. Minerva Anestesiol.

