# Supplementary figures and images for: Epidemiology, clinical characteristics, and outcome of candidemia in critically ill patients in Germany: a single-center retrospective 10-year analysis
Source: Ann Intensive Care. 2020 Oct 16;10:142. doi: 10.1186/s13613-020-00755-8 (PMC7567770; doi:10.1186/s13613-020-00755-8)

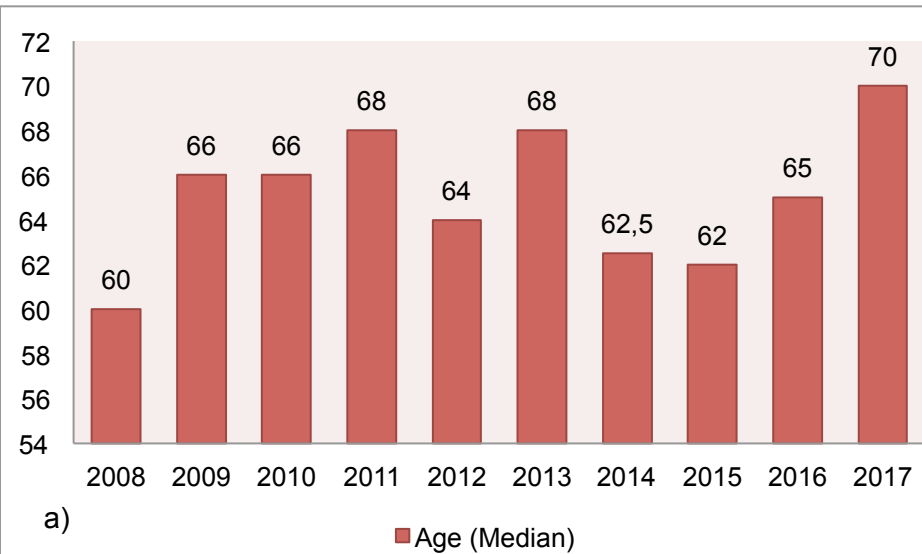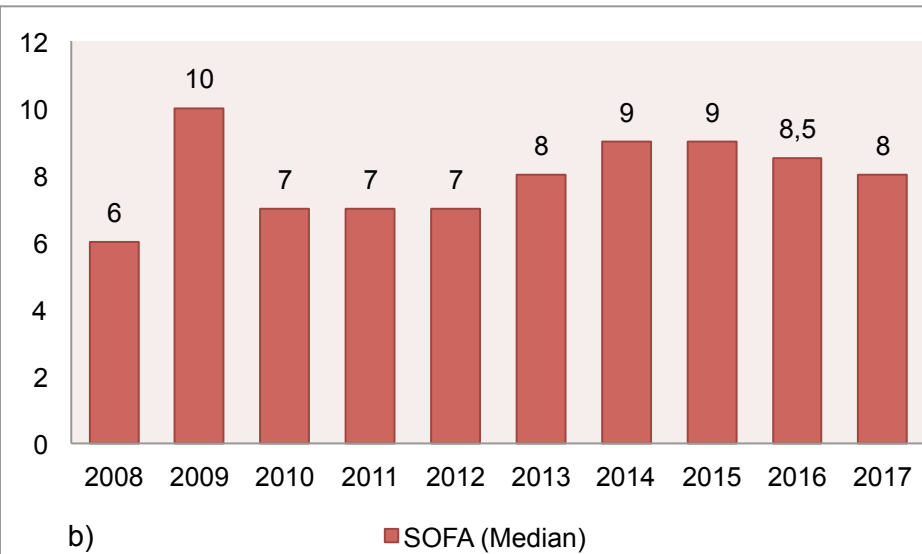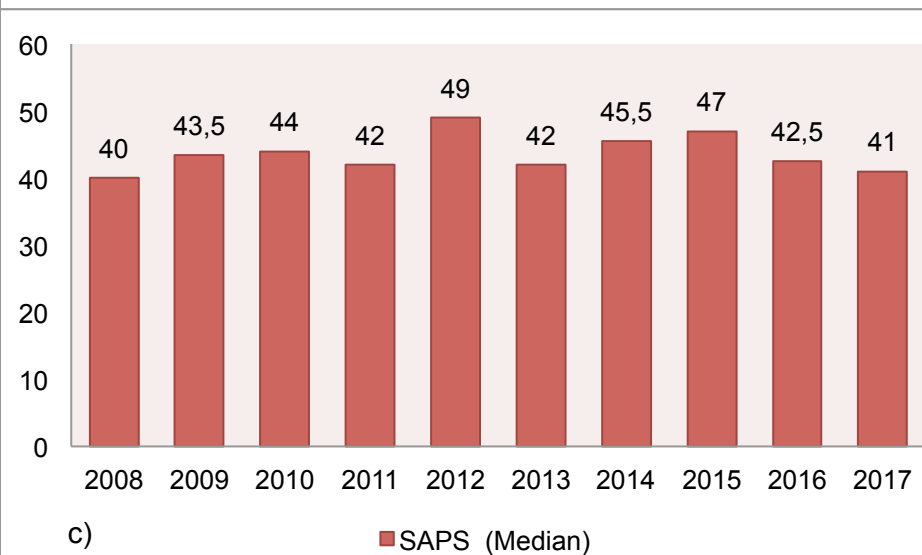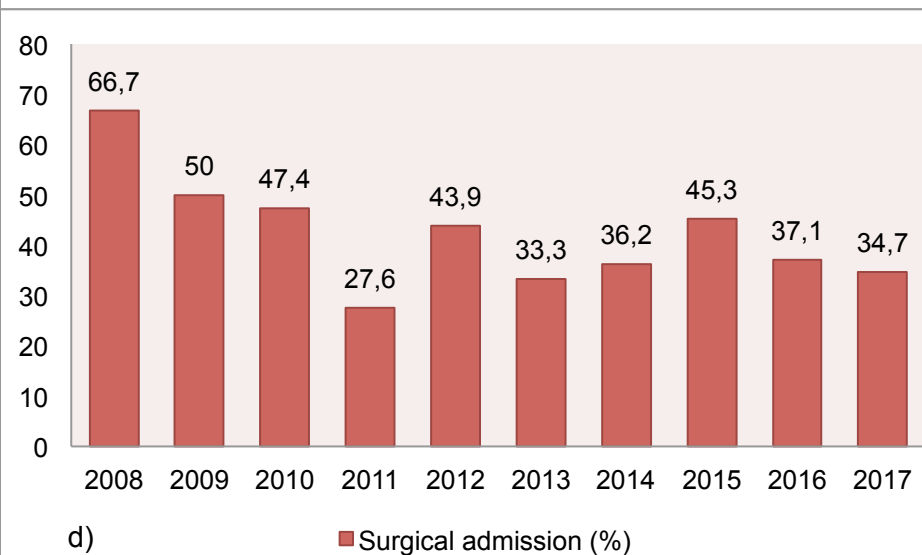

Supplement: Supplementary file 1 — Additional file 1. a-d: Selected patient characteristics throughout the study period between 2008 and 2017. a) Age, b) Sepsis-related Organ Failure Assessment (SOFA) score, and c) Simplified Acute Physiology Score on the day of Candida BSI are given as median. d) Surgical admission in %. [file 13613_2020_755_MOESM1_ESM.pdf]

# **Candida spp. in BSI patients n = 391**

☐ Survivors n = 157    ☐ Non-Survivors n = 234

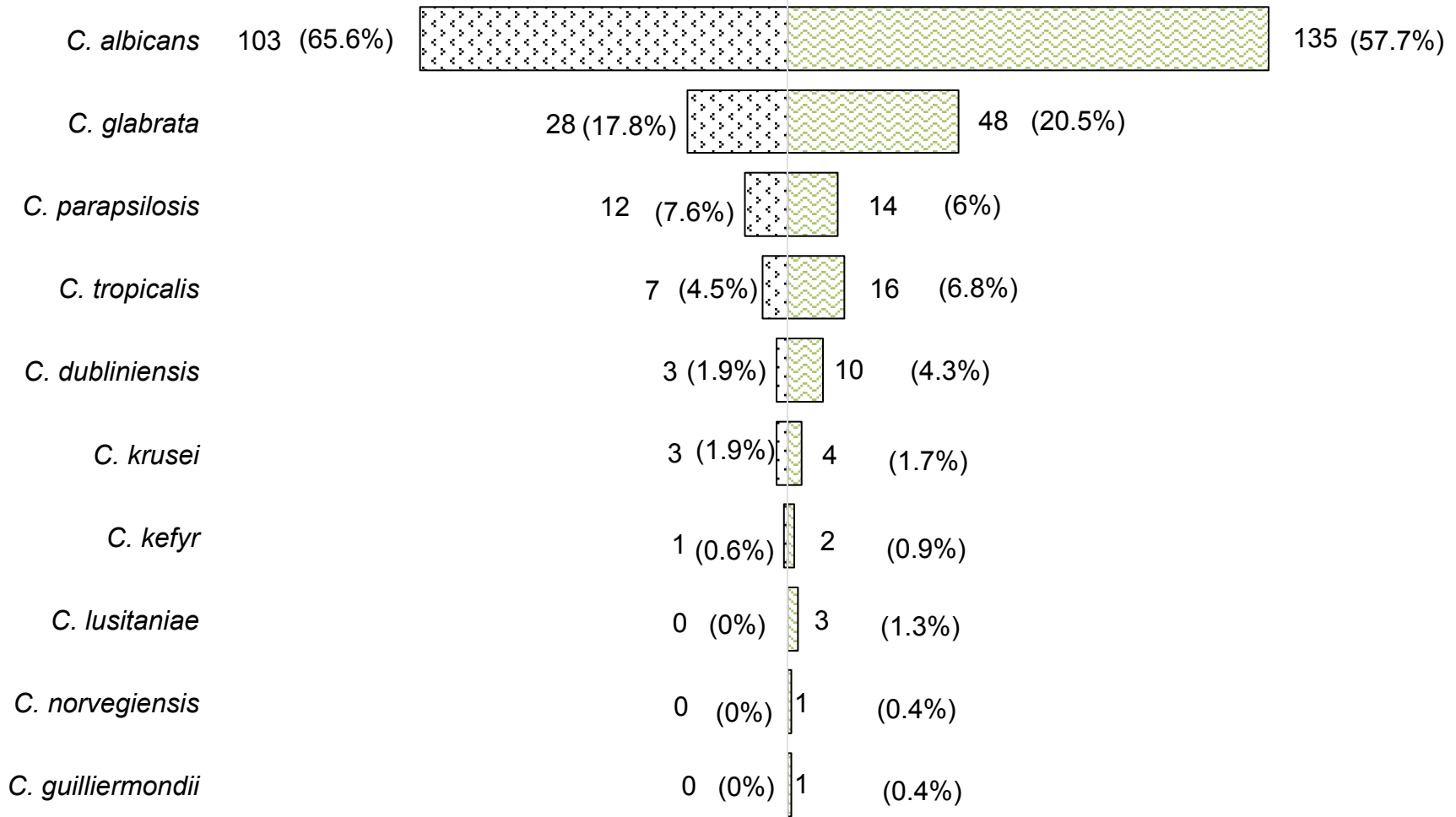

Supplement: Supplementary file 3 — Additional file 3. Distribution of Candida spp. in survivors (n = 157) and non-survivors (n = 234) at 180 days. BSI: bloodstream infection. [file 13613_2020_755_MOESM3_ESM.pdf]

# CANDIDA SPP. 2008 - 2017

■ albicans ■ non-albicans

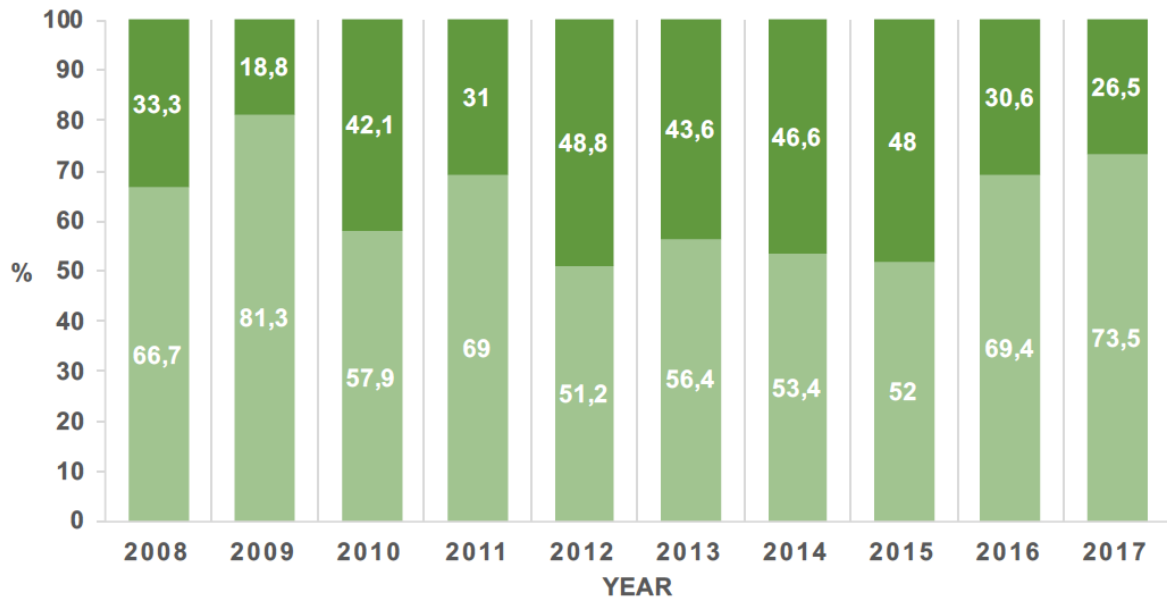

Supplement: Supplementary file 4 — Additional file 4. Distribution of C. albicans versus non-albicans Candida spp. between 2008 and 2017. [file 13613_2020_755_MOESM4_ESM.pdf]

## Factors associated with mortality 180 days after *Candida* BSI

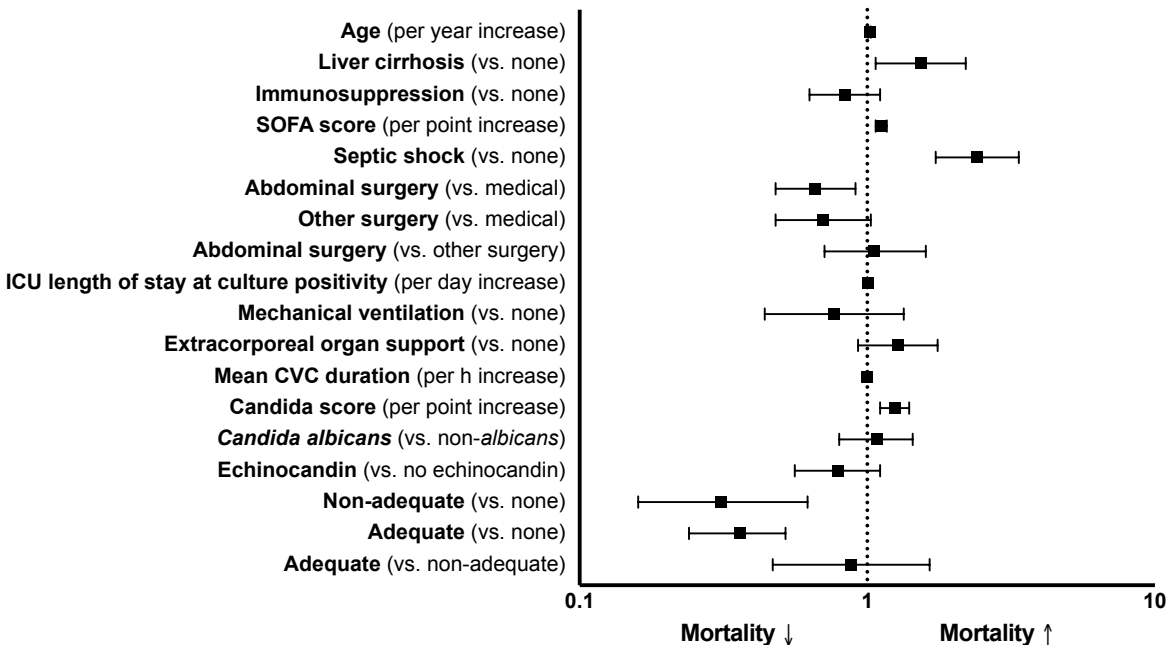

Supplement: Supplementary file 5 — Additional file 5. Predictors of mortality 180 days after blood cultures positive for Candida spp. were obtained. Squared symbols indicate hazard ratios, error bars indicate 95% confidence intervals. [file 13613_2020_755_MOESM5_ESM.pdf]
