# Supplementary material for: Epidemiology, clinical characteristics, and outcome of candidemia in critically ill patients in Germany: a single-center retrospective 10-year analysis
Source: Ann Intensive Care. 2020 Oct 16;10:142. doi: 10.1186/s13613-020-00755-8 (PMC7567770; doi:10.1186/s13613-020-00755-8)
Supplement: Supplementary file 2 — Additional file 2. Referring/co-managing specialties divided by mortality after 180 days. Data are given as absolute and relative numbers. aFisher’s exact test was performed for categorical variables. [file 13613_2020_755_MOESM2_ESM.pdf]

	Study participants n = 391	Non-survivors n = 234	Survivors n = 157	Descriptive p-value (unadjusted)[a]
**Co-managing specialty**				0.153
Visceral Surgery	121 (31)	69 (30)	52 (33)	
Gastroenterology and Hepatology	63 (16)	47 (20)	16 (10)	
Cardiology	35 (9)	21 (9)	14 (9)	
Pneumology	32 (8)	20 (9)	12 (8)	
Cardiovascular Surgery	21 (5)	13 (6)	8 (5)	
Trauma and Orthopedic Surgery	20 (5)	10 (4)	10 (6)	
Oncology, Hematology and Bone Marrow Transplantation	19 (5)	14 (6)	5 (3)	
Vascular Medicine	14 (4)	8 (3)	6 (4)	
Nephrology	13 (3)	5 (2)	8 (5)	
Visceral Transplant Surgery	12 (3)	7 (3)	5 (3)	
Neurology	10 (3)	6 (3)	4 (3)	
Neurosurgery	9 (2)	3 (1)	6 (4)	
Urology	9 (2)	3 (1)	6 (4)	
Otorhinolaryngology	6 (2)	2 (1)	4 (3)	
Gynecology	4 (1)	3 (1)	1 (1)	
Dermatology	3 (1)	3 (1)	0 (0)	
**Surgical vs. medical admission**				0.019
Surgery	216 (55)	118 (50)	98 (62)	
Conservative medicine	175 (45)	116 (50)	59 (38)	
